# Benefit of 3D Vena Contracta Area over 2D-Based Echocardiographic Methods in Quantification of Functional Mitral Valve Regurgitation

**DOI:** 10.3390/diagnostics13061176

**Published:** 2023-03-19

**Authors:** Vinzenz M. Jungels, Felix M. Heidrich, Christian Pfluecke, Axel Linke, Krunoslav M. Sveric

**Affiliations:** 1 Department of Internal Medicine and Cardiology, Herzzentrum Dresden, Technische Universität Dresden, Fetscherstr. 76, 01307 Dresden, Germany; 2 Department of Internal Medicine I, Städtisches Klinikum Görlitz, Girbigsdorfer Straße 1-3, 02828 Görlitz, Germany

**Keywords:** 3D echocardiography, functional mitral valve regurgitation, 3D VCA, 2D PISA

## Abstract

Background: The two-dimensional proximal isovelocity surface area (2D PISA) method in the quantification of an effective regurgitation orifice area (EROA) has limitations in functional mitral valve regurgitation (FMR), particularly in non-circular coaptation defects. Objective: We aimed to validate a three-dimensional vena contracta area (3D VCA) against a conventional EROA using a 2D PISA method and anatomic regurgitation orifice area (AROA) in patients with FMR. Methods: Both 2D and 3D full-volume color Doppler data were acquired during consecutive transoesophageal echocardiography (TEE) examinations. The EROA 2D PISA was calculated as recommended by current guidelines. Multiplanar reconstruction was used for offline analysis of the 3D VCA (with a color Doppler) and AROA (without a color Doppler). Receiver operating characteristic (ROC) analysis was used to calculate a cut-off value for the 3D VCA to discriminate between moderate and severe FMR as classified by the EROA 2D PISA. Results: From 2015 to 2018, 105 consecutive patients with complete and adequate imaging data were included. The 3D VCA correlated strongly with the 2D PISA EROA and AROA (r = 0.93 and 0.94). In the presence of eccentric or multiple regurgitant jets, there was no significant difference in correlations with the 3D VCA. We found a 3D VCA cut-off of 0.43 cm^2^ to discriminate between moderate and severe FMR (area under curve = 0.98). The 3D VCA showed a higher interobserver agreement than the EROA 2D PISA (interclass correlation coefficient: 0.94 vs. 0.81). Conclusions: The 3D VCA has excellent validity and lower variability than the conventional 2D PISA in FMR. Compared to the 2D PISA, the 3D VCA was not affected by the presence of eccentric or multiple regurgitation jets or non-circular regurgitation orifices. With a threshold of 0.43 cm^2^ for the 3D VCA, we demonstrated reliable discrimination between moderate and severe FMR.

## 1. Introduction

Functional mitral valve regurgitation (FMR) is associated with a worse outcome in patients with chronic heart failure [1]. The quantification of FMR is critical to identify patients who are suitable for and will benefit from interventional or surgical therapy. Echocardiography represents the main imaging modality for the visualization and quantification of FMR. Current guidelines recommend the effective regurgitation orifice area (EROA) according to a two-dimensional proximal isovelocity surface area (2D PISA) for the quantification of FMR [2,3]. Though there are many years of experience and ubiquitous availability of EROA 2D PISA, it has its drawbacks in FMR. The asymmetric, crescent-shaped regurgitation orifice in FMR poses a challenge for quantification using the conventional hemispheric 2D PISA method [4]. Recent studies showed that EROA 2D PISA underestimates the “real” regurgitation area due to the inadequate hemispheric assumption for asymmetric proximal convergence [5,6,7].

Therefore, new methods with direct measurement of the coaptation defect as the anatomical regurgitation orifice area (AROA) [8] or with measurement of the cross-section of the regurgitation jet, such as the three-dimensional vena contracta area (3D VCA) [9], were developed in the context of studies. However, in patients with FMR, there is limited data for these methods and no definite cut-off values for severity classification.

### The Aims of This Study

(1) Further substantiation of the measurement accuracy and reproducibility of 3D VCA in FMR;
(2) Additional proof for reliability of 3D VCA in situations with eccentric or multiple regurgitation jets;
(3) Determination of a cut-off value for 3D VCA to discriminate between moderate and severe FMR.

## 2. Materials and Methods

### 2.1. Study Population

**Figure 1:**
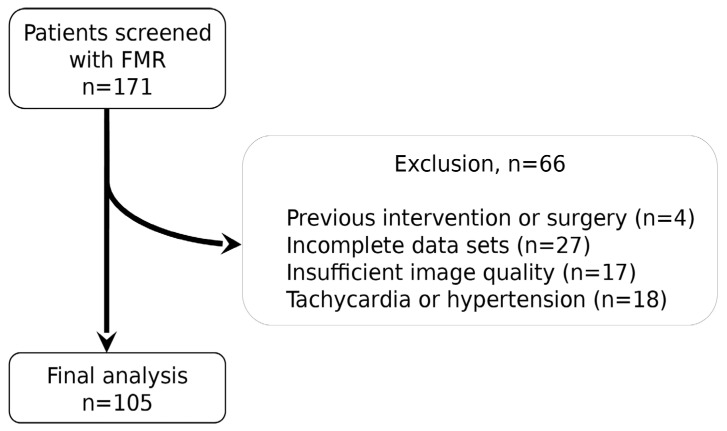
Screening procedure of patients with symptomatic moderate to severe FMR as part of the collective of patients enrolled in the “Dresden Mitral Valve Registry”. Abbreviation: FMR = functional mitral valve regurgitation.

Between 2015 and 2018, we screened 171 consecutive patients with moderate to severe FMR from the “Dresden Mitral Valve Registry”. This registry prospectively enrolls patients with symptomatic mitral regurgitation referred to our Department of Internal Medicine and Cardiology, Herzzentrum Dresden, Technische Universität Dresden, Germany, for further diagnosis and treatment. Exclusion criteria for analysis in this study were pre-existing mitral valve surgery or intervention, incomplete datasets, and insufficient image quality or clinical influencing parameters (Figure 1). Stitching artifacts in 3D datasets were the main cause of insufficient image quality in most cases (*n* = 13), while inadequate image acquisition affected fewer cases (*n* = 4) in both 3D and 2D.

### 2.2. Echocardiographic Parameters

Routine transesophageal echocardiographic examinations (TEEs) were performed after a standardized protocol by experienced examiners using a Philips IE-33 ultrasound machine equipped with a 3D matrix ultrasound probe (X7-2t). Offline measurements were performed using IntelliSpace Cardiovascular 3.1 (Philips Healthcare, Eindhoven, The Netherlands) and QLAB 10.5 (Philips Healthcare, Eindhoven, The Netherlands) software. In a 3D multi-beat volume of the left ventricle, left ventricular volumes and ejection fraction were determined according to the modified Simpson method. In the absence of a 3D dataset, left ventricular dimensions and function were obtained from pre-existing transthoracic echocardiographic images.

**Figure 2:**
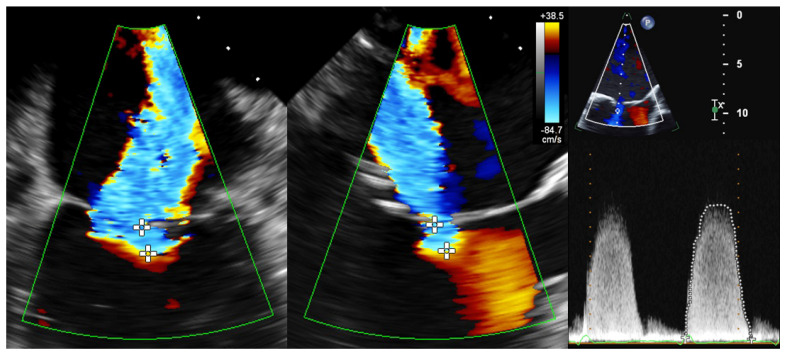
Determination of 2D PISA in biplane 2D color Doppler dataset and CW Doppler dataset. Echocardiographic images with 2 orthogonal slice planes of the 2D color Doppler dataset (mid-systolic) with Nyquist velocity of 38.5 cm/s (**left**); determination of PISA radius in 2 planes (diameters with crosses), MR-VTI (dotted outline), and MR-Vmax in the CW Doppler dataset (**right**) for calculation of regurgitation area. Abbreviations: 2D = two-dimensional; PISA = proximal isovelocity surface area; CW = continuous wave; MR-VTI = mitral regurgitation velocity–time integral; MR-Vmax = maximum mitral regurgitation velocity.

To determine the EROA using 2D PISA, biplane 2D color Doppler images (X-Plane views) were acquired optimally at a Nyquist velocity between 20 and 45 m/s. In the TEE 4-chamber view as well as in the orthogonal section plane with maximal expression of proximal convergence, the radius of proximal convergence between color change and plane of coaptation defect (minimal regurgitation area) was measured in mid-systole over different heartbeats. In addition, the maximum regurgitation velocity (MR-Vmax) and velocity–time integral (MR-VTI) in systolic phase of CW Doppler recordings were determined (Figure 2). Using the PISA method, the EROA was calculated according to the recommendations of the European Association of Cardiovascular Imaging (EACVI) [2].

**Figure 3:**
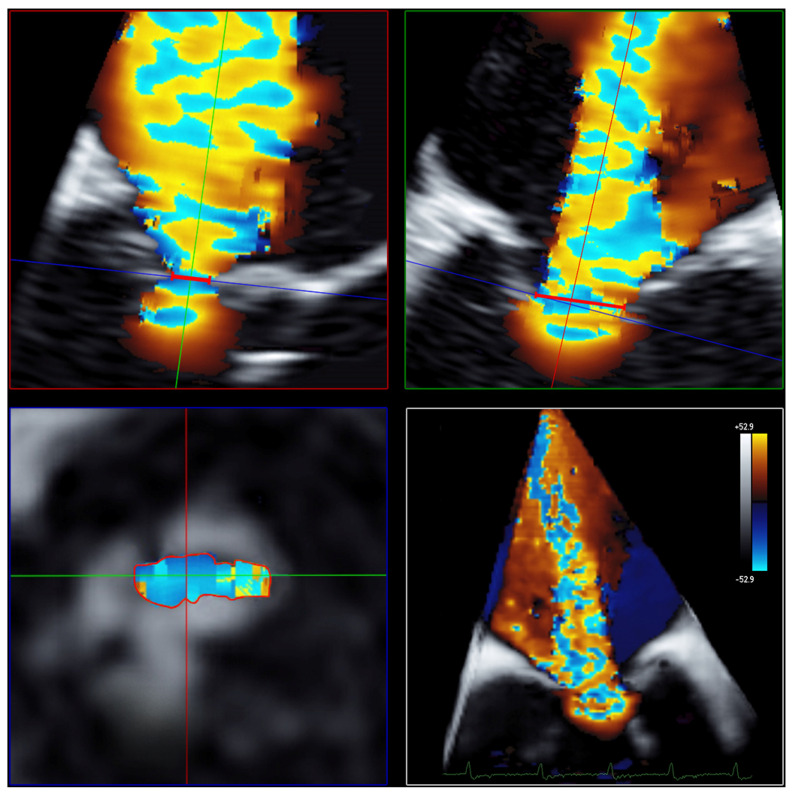
Determination of 3D VCA using multiplanar reconstruction in the 3D color Doppler dataset. Echocardiographic images with 3 slice planes and 3D reconstruction (**lower right**) of the 3D color Doppler dataset (mid-systolic). Planimetric fitting of the 2 upper planes along the course of the color Doppler jet; third plane (**lower left**) adjustment orthogonal to planes along the jet and finding the minimal cross-section of the regurgitation jet using translation and tilting of the third plane; manual measurement of the minimal cross-sectional plane using outlining. Abbreviations: 3D = three-dimensional; VCA = vena contracta area.

Measurements of 3D VCA and AROA were made using the full-volume 3D color Doppler datasets with the recommended limiting velocity of 50–70 m/s. The 3D VCA was planimetrically fitted to the minimum cross-sectional area of the regurgitation jet in the full-volume 3D color Doppler dataset (Figure 3). Considering the constraints of a curved plane, a manual measurement of the color Doppler of the cross-sectional plane was performed, excluding fractions of too-low velocities to determine the 3D VCA. Attention was paid to a constant setting of brightness and contrast and a fixed setting of color intensity at 50% values and smoothening.

In presence of multiple jets, the 3D VCA was measured for each jet individually, with optimal adjustment of the measurement plane to the respective single jet [10]. The total 3D VCA was determined as the sum of the areas of the individual jets.

**Figure 4:**
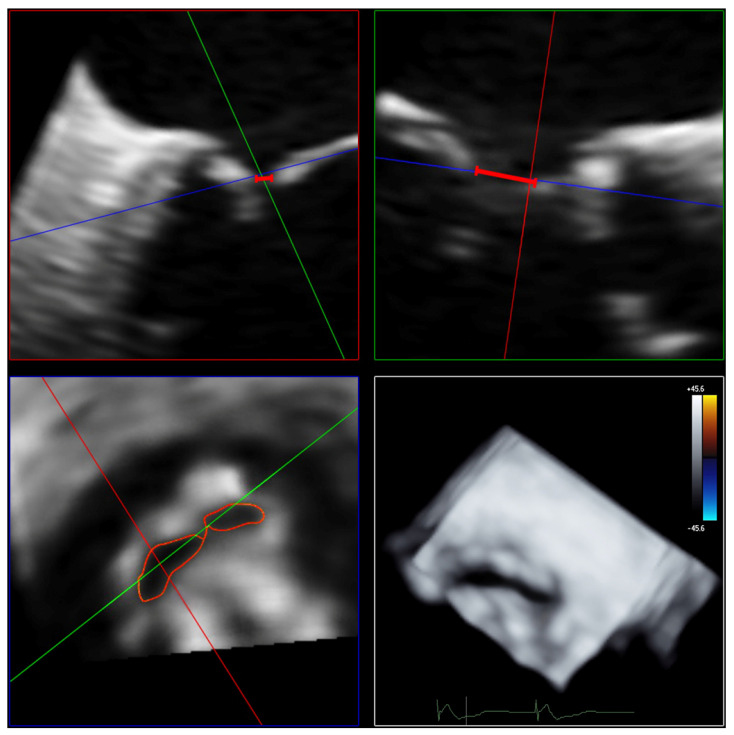
Determination of AROA using multiplanar reconstruction of the 3D color Doppler dataset without color. Echocardiographic images with 3 slice planes and 3D reconstruction (**lower right**) of the 3D color Doppler dataset (mid-systolic) with subtraction of color. Planimetric fitting of the 2 upper planes along the course of the color Doppler jet; third plane (**lower left**) adjustment orthogonal to planes along the jet. Then, subtraction of color and finding the minimum coaptation defect between the mitral valve leaflets using translation and tilting of the third plane; manual measurement of the minimum coaptation defect using outlining. Abbreviations: AROA = anatomical regurgitation orifice area; 3D = three-dimensional.

The AROA was measured by subtracting the color from the full-volume 3D color Doppler datasets. The AROA was planimetrically adjusted to the minimum cross-sectional plane of the anatomic regurgitation defect between the mitral valve leaflets (Figure 4). The area of the minimal anatomic regurgitation defect was measured by tracing the boundary of the leaflets. Analogous to the measurement of the 3D VCA, settings for brightness and contrast were fixed at 50% values and smoothening.

All measurements were performed as an average of triplicate determinations over several different heartbeats using recordings from different, mid-systolic heart phases.

### 2.3. Statistical Analyses

Statistical analyses were performed using Statistical Package for Social Science software (SPSS for Windows 22.0, Chicago, IL, USA). Significance level was set at *p* < 0.05. Continuous variables were evaluated using mean and standard deviation or median and interquartile range [Q1; Q3], as appropriate. Group comparisons were made using Student’s *t*-test or Mann–Whitney *U* test. For multiple groups, Kruskal–Wallis test was applied.

Correlations of the individual parameters were conducted using Pearson’s correlation procedure with indication of the associated correlation coefficient expressed as r. Comparison of correlations in different subgroups was conducted using Fisher’s *z*-test. Further comparison of the different methods was performed according to the recommendations given by Bland–Altman method, with the differences between two compared methods defined as bias, and associated tolerance ranges defined as the limits of agreement (LoA) were examined. Amount of proportional bias was assessed using linear regression analysis and expressed as regression coefficient R^2^.

Interobserver reliability was assessed using measurements of *n* = 20 randomly selected patients by an independent and blinded investigator (experienced senior cardiologist). As a result, interclass correlation coefficient (ICC) was calculated with 95% confidence interval (CI).

The receiver operating characteristic (ROC) was calculated using the EROA 2D PISA severity classification with a minimum cut-off value of 0.2 cm^2^ in accordance with European guidelines [2,11]. The area under the curve (AUC) was determined, and the optimal cut-off value was found using the Youden index (J) to maximize sensitivity and specificity.

## 3. Results

### 3.1. Characteristics of the Study Population

A total of 105 patients with symptomatic FMR were analyzed in this study. Male sex (66%) and higher age (median 79 years) were prevalent. Due to the functional origin of mitral regurgitation, most patients had a moderate to severely reduced left ventricular ejection fraction, and patients exhibited a dilated left ventricle (Table 1).

### 3.2. Echocardiographic Parameters

**Figure 5:**
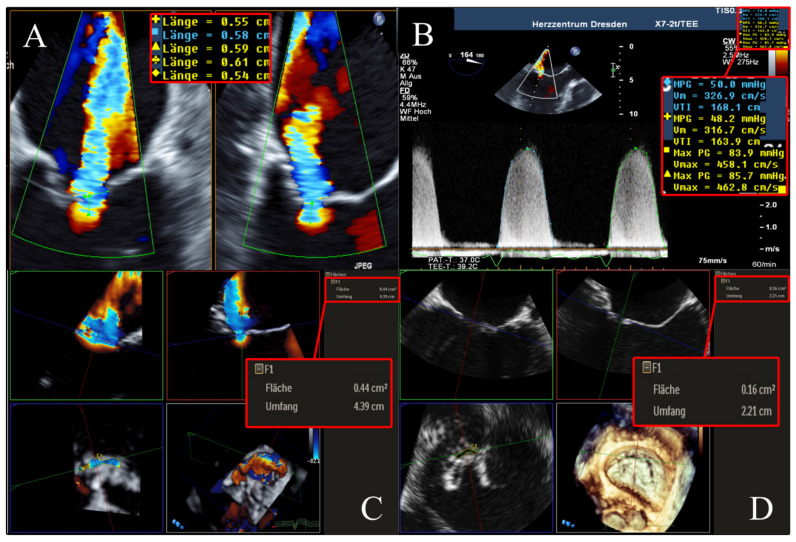
Example of a 51-year-old, male patient suffering from FMR due to non-ischemic dilated cardiomyopathy. (**A**,**B**) ERO quantified using 2D PISA method was 0.20 cm^2^, with a mean MR-Vmax of 460 cm/s. (**C**) 3D VCA derived by multiplanar reconstruction in a 3D color Doppler dataset was 0.44 cm^2^. (**D**) AROA derived by multiplanar reconstruction in a 3D Doppler dataset without color was 0.16 cm^2^. Abbreviations: FMR = functional mitral valve regurgitation; 2D = two-dimensional; PISA = proximal isovelocity surface area; CW = continuous wave; MR-Vmax = maximum mitral regurgitation velocity; 3D = three-dimensional; VCA = vena contracta area; AROA = anatomical regurgitation orifice area.

To facilitate a better understanding of our subsequent results of the cross-method comparisons, we illustrate in Figure 5 a representative example of the same patient evaluated for FMR using the 3D VCA, AROA, and EROA 2D PISA.

#### 3.2.1. Correlations and Bland–Altman Analysis of 3D VCA

**Figure 6:**
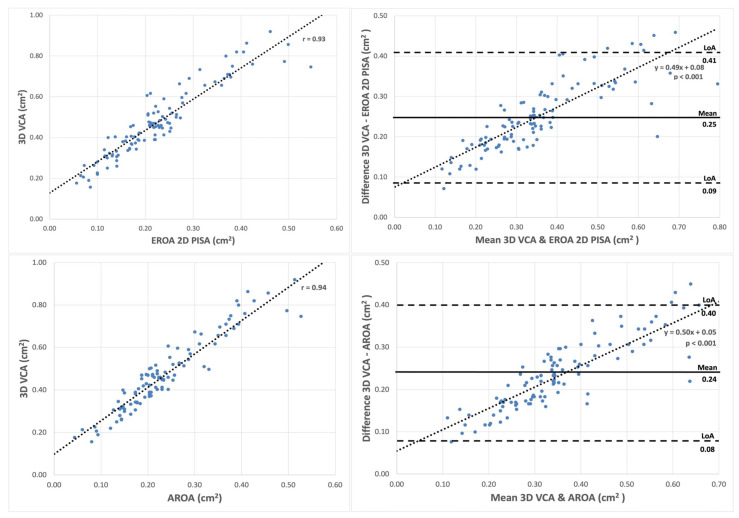
Correlations (**left**) and Bland–Altman plot (**right**) for 3D VCA. Correlation (**upper left**) and Bland–Altman plot (**upper right**) of 3D VCA compared to EROA 2D PISA. Correlation (**lower left**) and Bland–Altman plot (**lower right**) in comparison with AROA. Correlation plots with pairs of value using two methods. Bland–Altman plots with differences of two methods plotted against the mean values of the same methods (right). Dotted lines = fitting line of linear regression, solid lines = mean differences of two methods, dashed lines = limits of agreement (LoA). Abbreviations: 2D = two-dimensional; 3D = three-dimensional; EROA = effective regurgitation area; PISA = proximal isovelocity surface area; VCA = vena contracta area, AROA = anatomic regurgitation orifice area.

Although the 3D VCA correlated significantly with the EROA 2D PISA and AROA (r = 0.93 and r = 0.94, *p* < 0.001 for all), the 3D VCA comprised higher values than the EROA 2D PISA and AROA (0.47 ± 0.18 cm^2^ vs. 0.23 ± 0.11 cm^2^ vs. 0.24 ± 0.11 cm^2^, *p* < 0.001), resulting in a systematic measurement bias, as depicted in Figure 6. Mean bias between the 3D VCA and EROA 2D PISA (0.25 cm^2^; LoA: 0.09–0.41 cm^2^) and the AROA (0.24 cm^2^; LoA: 0.08–0.40 cm^2^) were statistically significant (*p* < 0.001 for all). In addition, the bias was proportional, with increasing amounts of regurgitation in both comparisons (R^2^ = 0.686 and 0.737, *p* < 0.001 for all).

Furthermore, the 3D VCA proved to have stable correlations with the EROA 2D PISA for situations with eccentric jets (r = 0.94) and multiple jets (r = 0.95). The mean bias between the 3D VCA and EROA 2D PISA was not affected by type (i.e., central vs. eccentric) or the amount of regurgitation jets (i.e., one vs. more) as follows: 0.26 ± 0.09 cm^2^ vs. 0.24 ± 0.07 cm^2^ vs. 0.25 ± 0.09 cm^2^ vs. 0.26 ± 0.08 cm^2^ (*p* > 0.18 for all).

#### 3.2.2. Threshold Determination for 3D VCA

**Figure 7:**
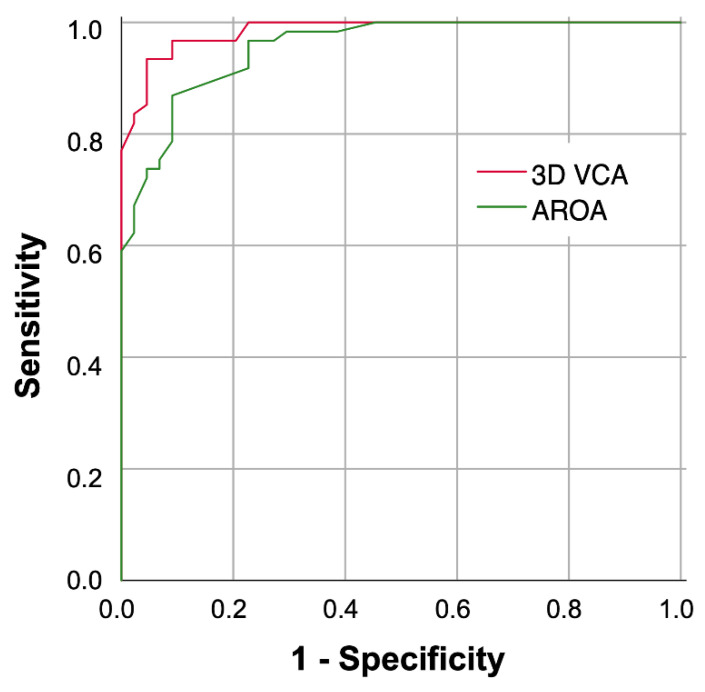
Receiver operating characteristic analysis of 3D VCA (AUC = 0.98) and AROA (AUC = 0.94). Graphical plotting of sensitivity and specificity using different methods to distinguish moderate and severe FMR classified by EROA 2D PISA (cut-off ≥ 0.2 cm^2^). Abbreviations: AUC = area under the curve; 3D = three-dimensional; VCA = vena contracta area; AROA = anatomic regurgitation area.

The ROC analysis, using a threshold value ≥ 0.20 cm^2^ for the EROA 2D PISA method for severe FMR, resulted in a cut-off value of 0.43 cm^2^ for the 3D VCA (J = 0.89), with an AUC of 0.98 (95%-CI: 0.97–1.00) and a sensitivity and a specificity of 93.4 and 95.5%, respectively (Figure 7).

#### 3.2.3. Reproducibility of Measurements

Based on the reproducibility analysis on 20 randomly selected patients, the 3D VCA (ICC = 0.94; 95%-CI: 0.76–0.99) and the AROA (ICC = 0.96; 95%-CI: 0.83–0.99) techniques comprised a higher interobserver agreement than the EROA 2D PISA method (ICC = 0.81; 95%-CI: 0.37–0.96).

## 4. Discussion

The main findings of our study are as follows:

(1) We demonstrated superior measurement accuracy and reproducibility of the 3D VCA compared to the 2D-based method in a collective, with a purely functional origin of mitral regurgitation;
(2) We showed that the 3D VCA is a robust parameter in challenging situations in FMR, especially in eccentric or multiple regurgitation jets;
(3) We established a cut-off value for the 3D VCA of 0.43 cm^2^ to differentiate between moderate and severe FMR, with high diagnostic quality.

The 3D VCA method has been developed to overcome and address the limitations of the 2D-based echocardiographic methods in measuring FMR. Several studies have shown that the conventional 2D PISA technique underestimates the true regurgitation area in FMR due to the inadequate hemispheric assumption of asymmetric proximal convergence [5,6,7]. In contrast to this, the 3D VCA directly assesses the crescent-shaped cross-sectional area of the regurgitation jet using planimetry and does not rely on geometric assumptions unlike the 2D-based method (Figure 3). Despite limitations, the 2D PISA was used as the reference method due to its widespread use, with many years of experience and the recommendations in the current guidelines [2]. In line with recent reports [4,5,8,12,13], we demonstrated the usefulness of the 3D VCA technique in quantifying FMR, especially in challenging situations, such as eccentric or multiple regurgitation jets [7,10].

However, an open question remains as to why the values for the 3D VCA were higher than for the EROA 2D PISA and AROA (i.e., the mean difference of 0.25 and 0.24 cm^2^). This would, at first sight, imply an overestimation of FMR severity using the 3D VCA method. Interestingly, our findings are in line with previous comparison studies on patients with mixed etiologies of mitral valve regurgitation, where the 3D VCA was also greater than the 2D-based standard method. Shanks and colleagues [7], as well Marsan and colleagues [14], demonstrated in their seminal studies that the 2D-based echocardiographic method systematically underestimates regurgitation volume and, therefore, underestimates regurgitation severity when compared to cardiac magnetic resonance imaging. The systematic bias of the 2D PISA method is attributed to an inadequate geometric approximation of the flow convergence resulting in a systematic underestimation of the regurgitation orifice area [7,14,15,16].

Not depending on geometric assumptions, AROA is a robust parameter, even in non-circular or multiple regurgitation orifices. We showed that AROA exhibits a good correlation with 3D VCA, which is in line with several previous studies [17,18]. However, the absolute differences in the comparison methods varied between the studies, which is likely due to different measurement methods. For AROA, we believe, that the systematic underestimation is due to difficulty in clearly defining the sails in cases of smaller coaptation defects. This needs to be elucidated in further studies.

Nevertheless, based on the recommended threshold value of 0.2 cm^2^ for the EROA 2D PISA method, we proved a reliable cut-off value of 3D VCA ≥ 0.43 cm^2^ for differentiation between moderate and severe FMR with a high diagnostic quality, which is in line with recent reports [6,15]. A further advantage of the 3D VCA technique is its high reproducibility. Our results showed excellent measurement reliability for the 3D VCA (ICC = 0.94), comparable to previous studies with ICC scores above 0.90 [6,7,13,14,15] and higher reliability than the 2D PISA (ICC = 0.81).

Our findings suggest that the 3D VCA method should be considered over the 2D PISA method for quantifying the severity of FMR. This is due to the 3D VCA’s use of planimetry and direct measurement of the cross-sectional area of the regurgitation jet rather than relying on geometric assumptions as the 2D PISA method does. We believe that our work contributes to the dissemination of the 3D VCA technique for routine clinical practice.

### Limitations

Finally, there are several limitations of this study that need to be addressed. As this study was performed at one center only, the external validity might be questionable. Multiple factors can cause wide variations in the outcome, for instance, differences in personnel and logistics in general and differences in TEE or TEE operator experience, in particular. However, there were only a limited number of highly qualified TEE operators following the same protocol during the examinations. Additionally, the data were re-evaluated by two blinded cardiologists, both experts in echocardiography. With the single-center character of the study, a selection bias cannot be ruled out. However, as the study population was an “all comers” selection, comprised patients with a very heterogeneous disease spectrum, this seems less likely. Differences in imaging quality were not addressed specifically. Image quality in the study population was generally good. Decreased image quality will most likely lead to different results. In these cases, an independent analysis method might prove to be superior to echocardiography.

## 5. Conclusions

Our results demonstrate the excellent validity and measurement reliability of the 3D VCA method in the evaluation of purely FMR. The 3D VCA is characterized by its robustness in dealing with eccentric or multiple regurgitant jets as well as non-circular regurgitant orifices in FMR. Although we have reproducibly established a cut-off value for 3D VCA to distinguish moderate from severe FMR with high diagnostic quality, further studies in larger cohorts are warranted.

## Figures and Tables

**Table 1:** Baseline characteristics of the studied population.

Characteristics	*n* = 105
Clinical:	
Age, years	79 [75; 83]
Male sex	69 (66)
BMI, kg/m^2^	25.8 [23.5; 29.6]
NYHA class	
II	31 (30)
III	58 (55)
IV	16 (15)
Systolic blood pressure, mmHg	106 [95; 125]
Heart frequency, beats/minute	76 [55; 85]
Etiology:	
Ischemic cardiomyopathy	62 (59)
Non-ischemic cardiomyopathy	37 (35)
Combined cardiomyopathy	6 (6)
Echocardiography:	
LVEDV, mL	157 [121; 198]
LVESV, mL	101 [62; 130]
LVEF, %	38 [25.5; 50.0]

Continuous variables are median with interquartile range [Q1; Q3], and categorical variables are absolute *n* (%). Abbreviations: BMI = body mass index, NYHA = New York Heart Association, LVEDV = left ventricular end-diastolic volume, LVESV = left ventricular end-systolic volume, LVEF = left ventricular ejection fraction.

## Data Availability

The data presented in this study are available upon request from the corresponding author. The data are not publicly available due to restrictions imposed by federal law.
